# Profile of Substance Use among Patients Attending De-Addiction Centres in a Coastal City of Southern India

**DOI:** 10.1371/journal.pone.0057824

**Published:** 2013-02-28

**Authors:** Nithin Kumar, Tanuj Kanchan, Bhaskaran Unnikrishnan, Rekha Thapar, Prasanna Mithra, Vaman Kulkarni, Mohan Kumar Papanna, Ramesh Holla, Saran Sarathy

**Affiliations:** 1 Department of Community Medicine, Kasturba Medical College, Manipal University, Mangalore, Karnataka, India; 2 Department of Forensic Medicine, Kasturba Medical College, Manipal University, Mangalore, Karnataka, India; 3 Kasturba Medical College, Manipal University, Mangalore, Karnataka, India; California Pacific Medicial Center Research Institute, United States of America

## Abstract

Drug dependence is still to be recognized in developing countries as a significant public health problem and literature on the magnitude of this problem is limited. The present research was planned to study the socio-demographic profile and the reasons for substance use among patients admitted at De-addiction centres in Mangalore, India. In this cross-sectional study, all the patients admitted at the De-addiction centres during the study period were interviewed. The data was analyzed and the results obtained were expressed in proportions. A total of 83 patients were included in the study, all of whom were males. A positive family history of substance use was evident in 63% of the respondents. The mean age of the study participants was 41.9 (SD±11.2) years and the mean age for starting substance use was 20.9 (SD±7.7) years. The most common substance used was alcohol (95.2%). Majority of the subjects (56.6%) cited peer pressure as a reason for initiating substance use. Our findings suggest that the initiation of substance use occurs during late teenage years and mostly due to peer pressure. Our observations point towards the vulnerability of younger age towards substance use and hence, it is proposed that the preventive health policies in this regard should be targeted specifically during teenage years.

## Introduction

The World Health Organization (WHO) defines Substance abuse as “Harmful or hazardous use of psychoactive substances, including alcohol and illicit drugs”. Repeated use of these substances can lead to dependence syndrome-a cluster of behavioral, cognitive, and physiological phenomena which involves a strong desire to take the drug, and difficulties in controlling its use [1]. Drug dependence poses not only economic burden through rising health care costs, but also social costs in the form of loss of productivity and family income, violence, security problems, traffic and workplace accidents [2]. With most drug users being in 18–35 years age group, the loss in terms of human potential is incalculable. Adolescent drug abuse is one of the major areas of concern in adolescent and young people's behavior [3].

Drug dependence is still to be recognized as a significant public health problem in India. Though it is a preventable and treatable disease with effective prevention and treatment interventions, people suffering from it are stigmatized and many a times have no access to treatment and rehabilitation. The WHO reports that the main location for treatment of substance use disorders is the specialist substance abuse system, followed by the mental health system, the general health system, and primary care [1]. Considering the current magnitude of substance use in most societies, and the limited resources available for treatment there is a need to develop services that can reach the maximum number of individuals and have the greatest impact at lowest cost. This can be achieved with broad community-based health care services that can work with individuals in their own communities over longer periods of time [2].

Though the problem of substance abuse has been prevailing in our country for quite some time, the magnitude of the problem is never reflected or highlighted in the official survey data or statistics [4]. The data on the problem status of substance use in the study region itself is limited. The present research is an attempt to study the socio-demographic profile of patients attending De-addiction centres in Mangalore to find the various substances commonly used by them along with the reasons for driving them to resort to the substance use and falling prey to this serious public health problem.

## Patients and Methods

Ethics Committee approval was obtained from the Institutional Ethics Committee of Kasturba Medical College, Mangalore (affiliated to Manipal University), India prior to the commencement of the study. Permissions were sought from the authorities in charge of the individual De-addiction centres and subsequently three De-addiction centres were included in the study. Two of the three De-addiction centres included in the study are run by Non-governmental Organizations (NGOs) while the third one is managed by a charitable institution. These centres have both in-patient and out-patient programs. However, the present study was conducted on the in-patients only. The patients were approached individually and explained about the objectives of the study in their preferred language. A written informed consent was taken from the patients who were willing to participate in the study. All the participants were then interviewed.

The present cross-sectional study was conducted in Mangalore, a coastal city in South India during April 2012. All the patients included in the study were interviewed during the study period. Information pertaining to the study variables was collected using a semi-structured questionnaire, which was divided into three sections. Section A collected information on socio-demographic variables, Section B on the type of substances used, duration of use, and reasons for its use, and Section C on the treatment details. During the interview, the participants were asked to respond to the questions and all their responses were marked by the interviewer. The data thus, collected was analyzed using SPSS (Statistical Package for Social Sciences) version 11.5 and the results obtained were expressed in proportions.

### Details of Ethical Approval

Ethics Committee approval was obtained from the Institutional Ethics Committee of Kasturba Medical College, Mangalore (affiliated to Manipal University), India prior to the commencement of the study.

## Results

A total of 83 patients were interviewed in the De-addiction Centres during the study. All the patients admitted at the De-addiction centres were males. The mean age of the patients was 41.9 (SD ±11.2) years with nearly 1/3^rd^ of the patients (31.3%) were aged between 31 and 40 years. A large proportion (39.8%) of the patients had completed their graduation and 48.2% were involved in skilled occupation. The majority of the patients were married (74.7%) and a considerable proportion (77.0%) belonged to nuclear families. A high percentage of patients (63.0%) had a history of substance use in the family. Socio-demographic information of the patients is detailed in Table 1.

**Table 1:** Socio-demographic information of the patients (n = 83).

Socio-demographic variables	n (%)
**Age group**	
<20	01 (01.2)
21–30	14 (16.9)
31–40	26 (31.3)
41–50	23 (27.7)
>50	19 (22.9)
**Educational status**	
Illiterate (No formal education)	03 (03.6)
Primary (Schooling up to class 8)	10 (12.0)
Secondary (Schooling up to class 10)	21 (25.3)
Higher secondary (Schooling up to class 12)	11 (13.3)
Graduation (Bachelor’s degree)	33 (39.8)
Post-graduation (Master’s degree)	05 (06.0)
**Occupational status**	
Unskilled	29 (34.9)
Skilled	40 (48.2)
Professional	12 (14.5)
Others	02 (02.4)
**Marital status**	
Married	62 (74.7)
Unmarried	18 (21.7)
Divorced	02 (02.4)
Separated	01 (01.2)
**Type of family**	
Nuclear	64 (77.0)
Joint	16 (19.0)
Extended	03 (04.0)
**Family History of substance use**	
Yes	52 (63.0)
No	31 (37.0)

Information on the age of initiation, duration of exposure and types of substances used are shown in Table 2. The mean age for initiation of substance use was 20.9 (SD ±7.7) years. A majority of patients started using substances before the age of 20 years (61.4%) and none initiated the use of substances beyond the age of 50 years. It is observed that with increasing age there was a decrease in number of individuals initiating substance use. The mean duration of substance use was 19.1 (SD ±11.7) years. Most of the patients were single or mono substance users (55.5%) and the remaining (44.5%) were poly-substance users. The most common route of substance use was oral, followed by inhalation, nasal and intravenous route. Alcohol was the most common substance (95.2%) used by the patients, either singly, or in combination with other substances.

**Table 2:** Age of initiation, duration, and types of substances used (n = 83).

Characteristics	n (%)
**Age of initiation of substance use (years)**	
≤20	51 (61.4)
21–30	22 (26.5)
31–40	07 (08.4)
41–50	03 (03.6)
**Duration of substance use (years)**	
≤10	26 (31.3)
11–20	26 (31.3)
21–30	15 (18.1)
31–40	13 (15.7)
>40	03 (03.6)
**Type of substances used**	
Alcohol	79 (95.2)
Tobacco	30 (36.1)
Heroin	04 (04.8)
Cannabis	01 (01.2)
Prescription drugs	01 (01.2)

Reasons for initiating and continuing substance use are detailed in Table 3. Most of the patients (56.6%) reported that peer pressure was a major influence for taking up substance use. Work and family related stress was the most common reason (54.2%) for the regular use of substances among patients. 59.0% patients realized that they were dependent on the substances owing to their continuous craving for the same.

**Table 3:** Reasons for initaiating and continuingsubstance use (n = 83).

Reasons for initiating substance use	n (%)
Peer pressure	47 (56.6)
Curiosity	28 (33.7)
Family related stress	15 (18.1)
Work related stress	08 (09.6)
Academic stress	03 (03.6)
Role models	02 (02.4)
**Reasons for regular use of substances**	**n (%)**
Family related stress	23 (27.7)
Work related stress	22 (26.5)
Experiencing withdrawal features	13 (15.7)
Easy accessibility	13 (15.7)
Low self esteem	10 (12.0)
Enjoyment	08(09.6)
**Reasons leading to realization of dependence**	**n (%)**
Continuous craving	49 (59.0)
Inability to do productive work	16 (19.3)
Self-realization	16 (19.3)
Started experiencing medical problems	10 (12.0)
Made aware by the family members	03 (03.6)

Counseling was the most common form of treatment given to these patients (89.2%), followed by detoxification (44.6%) and behavioral therapy (10.8%). Majority of the patients (79.5%) were brought to the De-addiction centres by their family and friends while 19.3% had approached the De-addiction centres on their own.

## Discussion

India is facing a problem of multiple epidemics with infectious diseases at one end of the spectrum and life-style related diseases at the other end. The diseases affecting mental health, including substance abuse disorders constitutes the remainder of the spectrum. In the present study, all the patients who attended the De-addiction centres were males. While females are largely confined to indoors, the males have a more easy access to illicit substances than females. The lack of female patients at the De-addiction centres can also be attributed to the poor health seeking behavior among females, owing to the embarrassment and shame they might face on revealing this behavior to their families and society. Studies from the South East Asia Region have also reported a male predominance [5]–[7].

The majority of the patients were below 30 years of age. Studies from Pakistan [5], [6] and India [8] have reported similar observations in this regard. A study from Ahmadabad,India [9] observed 46% patients to be below 20 years of age. Educational and employment status of the patients is shown to vary in studies from different regions. In the present study, 39.8% patients had completed their graduation. In Ahmadabad, India [9] 39.1% patients had completed their higher secondary education whereas in Ghaziabad, India [8] 40.3% patients had completed their primary education only. None of the patients in our study were unemployed prior to getting admitted to the centre and nearly half of them (48.2%) were involved in skilled work. In Karachi, Pakistan [6] 29.6% patients were unemployed; whereas in Chennai, India [10] 31.7% were unemployed. A very small number of patients in our study (3.6%) were either divorced or separated which is significantly lower to that reported in a study from Singapore [7] where 22.3% patients were divorced or separated. The presence of substance use in the family seems to be a significant factor in our study when compared to studies from Ghaziabad [8] and Ahmadabad [10], where family history of substance use was observed in 24.8% and 26% patients respectively.

The mean age for the initiation of substance use in our study was 20.9 years which is very similar to that reported in a study from Iran [13]. A comparatively lower mean age of initiation of substance use was reported in studies from Chennai [12] and Faridkot [14] in India, where the mean age was 17.7 years and 15.0 years respectively. The mean duration of substance use in our study was 19.1 years which is higher to that reported in a study from Iran [11]. In our study, majority of the patients cited peer-pressure as a reason for initiating substance use.

Peer pressure refers to the influence of peer group on an individual to act and think in certain manner irrespective of individual’s personal wishes [13]. Peer pressure can lead to a positive or a negative choice and practice. A significant association of peer pressure with use of drugs and alcohol is well established [14]. Borsari and Carey [15] observed that the peer environment contributes to high-risk alcohol use by way of direct influences, modeling, and perceived norms. Kobus [16], in a comprehensive review observed that adolescent peer relationships contribute to adolescent cigarette smoking. In a study from Chandigarh [17], peer pressure was the single most important cause for initiation of substance use whereas in a study from Kashmir [18], 44.4% patients were influenced by peer group to use substances. Our findings in this regard are in agreement with that reported in earlier studies.

In a study from Ahmadabad [9], 70.2% of the patients were addicted to alcohol which is similar to our study findings. The global burden of disease attributable to alcohol and illicit drug use amounts to 5.4% of the total burden of disease [1]. The National Family Health Survey 3 (NFHS-3) data from India showed that in the age group of 15–24 years, the prevalence of alcohol consumption was 1% among females and 19% in males [19]. As per the World Drug Report, Cannabis is the most widely used illicit drug followed by Amphetamine-type stimulants, opioids and cocaine. However, there is a lack of information regarding use of illicit drugs in countries such as China and India, as well as in emerging regions of consumption [20].

Initiation of substance use occurs during late teenage years which points towards the vulnerability of individuals towards substance use and addiction. A considerable number of patients were resorting to poly substance use which can be attributed to the easy accessibility and availability of these substances especially alcohol and tobacco products. Community based studies need to be conducted to estimate the magnitude of this problem and to study the conditional factors influencing the use of substances especially among the adolescents. Health education regarding the harmful effects (physical and psychological) of substance use should be addressed in schools and colleges. Awareness Programs and Camps need to be conducted at community level to address this major public health problem of substance use.
